# Sex Differences in Leptin Levels in Children and Adolescents with Normal Weight and Overweight/Obesity Across Pubertal Stages: A Systematic Review and Meta-analysis

**DOI:** 10.1210/clinem/dgaf621

**Published:** 2025-11-15

**Authors:** Rutuja Dhamale, Alejandra V Rodríguez Rondón, Eline E P L van der Walle, Erica L T van den Akker, Jenny A Visser

**Affiliations:** Obesity Center CGG and Center of Expertise for Genetic Obesity, Erasmus MC, University Medical Center Rotterdam, 3000 CA Rotterdam, the Netherlands; Department of Internal Medicine, Division of Endocrinology, Erasmus MC, University Medical Center Rotterdam, 3000 CA Rotterdam, the Netherlands; Obesity Center CGG and Center of Expertise for Genetic Obesity, Erasmus MC, University Medical Center Rotterdam, 3000 CA Rotterdam, the Netherlands; Department of Internal Medicine, Division of Endocrinology, Erasmus MC, University Medical Center Rotterdam, 3000 CA Rotterdam, the Netherlands; Obesity Center CGG and Center of Expertise for Genetic Obesity, Erasmus MC, University Medical Center Rotterdam, 3000 CA Rotterdam, the Netherlands; Department of Pediatrics, Division of Endocrinology, Erasmus MC-Sophia Children's Hospital, University Medical Center Rotterdam, 3000 CA Rotterdam, the Netherlands; Obesity Center CGG and Center of Expertise for Genetic Obesity, Erasmus MC, University Medical Center Rotterdam, 3000 CA Rotterdam, the Netherlands; Department of Pediatrics, Division of Endocrinology, Erasmus MC-Sophia Children's Hospital, University Medical Center Rotterdam, 3000 CA Rotterdam, the Netherlands; Obesity Center CGG and Center of Expertise for Genetic Obesity, Erasmus MC, University Medical Center Rotterdam, 3000 CA Rotterdam, the Netherlands; Department of Internal Medicine, Division of Endocrinology, Erasmus MC, University Medical Center Rotterdam, 3000 CA Rotterdam, the Netherlands

**Keywords:** leptin, puberty, sex differences, sexual maturation, obesity

## Abstract

**Context:**

Leptin levels differ significantly between adult men and women, it is unknown whether these sex differences arise during puberty in children with normal weight (NW) or overweight (OW)/obesity (OB).

**Objective:**

To analyze sex differences in leptin levels and body mass index SD score across pubertal stages in children with NW and OW/OB.

**Data Sources:**

Eligible studies were obtained from Medline, Embase, Web of Science, Cochrane, and CINAHL from inception until February 2025.

**Study Selection:**

Twenty-four of 1713 studies assessing leptin levels in children aged 5 to 19 years were included.

**Data Extraction:**

Two reviewers independently extracted data and assessed study quality.

**Data Synthesis:**

We performed subgroup meta-analysis stratified for pubertal stage using random effects model to estimate the weighted mean difference in R. Girls with NW had higher leptin levels than boys at all pubertal stages (pooled mean difference [MD]: 3.99; 95% CI, 2.63-5.35). In children with OW/OB, no significant differences were found in the prepubertal and pubertal stages. At the postpubertal stage, leptin levels were higher in girls compared to boys (MD: 14.60; 95% CI, 0.95-28.25), based on 1 included study. In pubertal children with OW/OB, body mass index SD score was higher in boys than girls (MD: −0.67; 95% CI, −0.74 to −0.61).

**Conclusion:**

The sex-specific differences in leptin levels, characteristic of normal-weight (pre-)pubertal children is lost in obesity. Therefore, leptin levels alone are unlikely to explain why obesity accelerates puberty in girls more than in boys. A combined effect with other factors, such as sex dimorphism in kisspeptin, may play a role.

Obesity (OB) is a major metabolic concern affecting the physiological and psychosocial aspects of the pediatric and adult population. The prevalence of OB among children and adolescents has risen dramatically in recent years. Childhood overweight (OW)/OB are defined as a body mass index (BMI) above the 85th and 95th sex- and age-specific percentiles or +1 and +2 BMI-SD score (SDS), which have been smoothed by International Obesity Task Force, resulting in age- and sex-specific BMI cutoffs (1, 2). Obesity is a complex pathophysiological disease caused by an excess and dysfunctional adipose tissue (3). The adipokine leptin is produced by adipocytes and levels are elevated in obesity (4). Leptin is a multifunctional neuroendocrine peptide hormone and plays a major role in the regulation of energy homoeostasis through the leptin-melanocortin pathway in the hypothalamus (5). In addition, leptin is known to have other actions, including on reproduction and sexual maturation in children. In particular, leptin plays a permissive role in the onset of puberty (6). Animal studies suggest that leptin administration causes onset of puberty in female mice (7). In humans, congenital leptin deficiency or pathological conditions like anorexia nervosa characterized by low leptin levels cause hypogonadotropic hypogonadism by failing to activate the hypothalamus-gonadal axis and failure of pubertal initiation (8). This suggests that optimal leptin levels are necessary for the initiation of puberty.

Leptin is secreted by the adipose tissue when the body is in a well-fed condition, thereby indicating caloric sufficiency. Leptin secretion decreases in times of fasting, which indicates caloric deprivation. Upon reaching a state with sufficient body fat to maintain threshold levels of leptin, leptin is thought to contribute to the maturation of the hypothalamus-pituitary-gonadal (HPG) axis. It stimulates the hypothalamus to secrete GnRH, which subsequently results in the release of the pituitary gonadotropins, LH and FSH. The effects of leptin on the HPG axis are regulated by a complex neural system consisting of kisspeptin, neurokinin B, and dynorphin neurons and pro-opiomelanocortin (POMC) neurons present in the arcuate nucleus of the hypothalamus (8, 9). Thus, leptin not only acts as a connecting link between the metabolic and the reproductive axis but also acts as a permissive gate in the initiation of puberty (8).

Puberty is a temporal cascade of biological events and normally begins at the age of 8 to 13 years in girls and 9 to 14 years in boys. Pubertal development, marked by the activation of HPG-axis that stimulates the release of the sex steroids estrogen (in girls) and testosterone (in boys), is assessed according to the Tanner stages (10). Tanner stage 1 represents the prepubertal stage in both boys and girls. In girls, Tanner stage 2 is described by development of breast buds, known as thelarche. This is followed by breast development at Tanner stage 3, and finally progresses to menarche, which marks Tanner stage 4 or the postpubertal stage. In boys, the increase in testicular volume by >4-mL marks the beginning of puberty (Tanner stage 2), followed by genital development and hoarseness of voice at Tanner stage 3. The final stage, Tanner stage 4, is known as spermarche (ie, development of sperm in males), also denoted as the postpubertal stage (10). The pubertal process is regulated by many factors, but the amount of body fat plays a crucial role. Numerous studies suggest that leptin drives the connection between body fat and puberty timing (11). It is known that girls with a higher BMI are prone to precocious (ie, early) puberty (11). Longitudinal studies have suggested that girls with an earlier onset of obesity are more likely to have an earlier pubertal onset compared to their peers (12, 13). Studies conducted in various countries have confirmed that a higher BMI-SDS in girls is associated with an earlier breast bud development and earlier menarche (14, 15). However, BMI does not seem to influence pubertal timing in boys in a similar way; studies show conflicting results (16, 17). Research suggests that obesity exerts sex-specific effects on pubertal onset, potentially through leptin regulation (18). Moreover, in contrast to girls, pubertal boys with OB tend to have lower testosterone levels than normal weight (NW) peers (19). This raises the question of whether this difference in pubertal timing between girls and boys can be explained by sex-specific changes in leptin levels during puberty.

Although no significant sex differences are observed in body fat distribution until early puberty, the sexual dimorphism in sex steroids and fat composition, particularly in fat mass and fat-free mass, becomes evident during pubertal development (20). In the later stages of puberty, a continuous increase in testosterone and fat-free mass along with a decrease in fat mass is observed in boys. In contrast, an increase in estrogen and fat mass with a reduction in fat-free mass is observed in girls (21). Thus, as a function of adiposity and fat mass, sex differences are also observed in leptin levels across pubertal stages (22). However, in children with OB, the overall increase in body mass makes it difficult to determine whether the typical sex differences in fat distribution and consequently, leptin levels, still persist. Therefore, we aimed to conduct a systematic review and a meta-analysis to evaluate sex differences in leptin levels during puberty in children and adolescents with NW compared to OW/OB. Along with leptin, we analyzed sex differences in BMI-SDS to better understand differences in body composition in boys and girls during puberty in both NW and OW/OB groups.

## Methods

### Search Strategy and Inclusion and Exclusion Criteria

The search strategy was developed in Embase by the Medical Library at Erasmus MC, Rotterdam, using the following key words: “puberty” OR “sexual development” OR “leptin” OR “menarche” OR “adrenarche” OR “sex differences” OR “estrogen” OR “testosterone” OR “girl” OR “boy”. This was also applied to other databases such as Medline, Web of Science, Cochrane, and CINAHL (Table S1) (23). We searched for articles from inception until March 17, 2025. The retrieved articles were imported in Covidence (www.covidence.org). Studies were included if they were a case-control study, population cohort, or an observational study. Studies performed on adults and animals and in vitro studies as well as other types of narrative reviews and systematic reviews were excluded. Furthermore, the study population should include children and/or adolescents evaluating the following outcomes: weight category, leptin levels, and pubertal/Tanner stages. Studies were excluded if it they were randomized trials or involved study populations analyzing gene polymorphisms; if the sample was not generalizable to the general population (eg, presence of comorbidities), or if they focused on interventions (lifestyle or medication), intrauterine exposures, or other forms of exposures such as parental smoking or exposure to endocrine disrupting chemicals such as pesticides. Studies performed in athletes, swimmers, or any form of exercise were also excluded. These articles were excluded to avoid confounding effects on leptin concentrations and to be able to generalize the results to the general population. When data of the same participants were included in 2 or more studies by the same author, the study with the larger dataset was included.

### Data Extraction and Risk of Bias Assessment

A data registration form was created in Covidence according to the following variables of interest: sample size, sex, age, Tanner stages, leptin levels (ng/mL), and BMI-SDS reported in both girls and boys distinguishable by the author's surname and the publication year. The form was optimized while accommodating for these variables with the help of 2 reviewers (R.D. and A.R.). The retrieved articles were screened for title and abstract based on the following criteria: studies performed on children and adolescents between 5 to 19 years of age and studies that evaluated leptin as an outcome. Next, full-text screening was performed while taking the inclusion and exclusion criteria into account. Discrepancies in results were resolved by a third reviewer (J.V.). The selected articles were clustered into 2 groups: (1) children with NW and (2) children with OB/OW (BMI >85th percentile for OW and BMI >95th percentile for OB), and the study information was recorded accordingly. The extracted data were also included in a spreadsheet. To assess the quality of the selected studies, Newcastle-Ottawa scale for observational studies was used. This scale assesses the quality of the included articles based on selection of the groups, comparability, and ascertainment of outcome in cohort studies and exposure in case-control studies. The quality assessment was performed individually by 2 reviewers (R.D. and A.R.) and discrepancies were discussed. A score >6 was considered high quality (Tables S2A–C) (23). No studies were excluded based on quality.

### Statistical Analysis

The statistical analysis was performed with R-studio (version 4.1.2) using meta, metadata, and metaphor packages. To estimate sex differences in leptin levels, leptin concentrations were converted to mean ± SD where necessary. In studies that reported leptin values as median and interquartile ranges, we used the methodology of McGrath et al to estimate the mean and SD (24). In cases where SEs and CIs were reported, we used the standard equations to estimate the SD. The meta-analysis was stratified by pubertal stages (prepubertal, pubertal, and postpubertal). In studies where leptin levels were reported according to Tanner stages, we reclassified the Tanner stages to pubertal stages as follows: Tanner stage 1 was redefined to prepubertal, Tanner stages 2 and 3 were combined to pubertal, and Tanner stages 4 and 5 to the postpubertal stage. The mean leptin levels at these stages were combined using the weighted mean equation. To increase the number of studies in the meta-analysis, we included the control groups from the case-control studies in the NW group for studies if they reported BMI/BMI-SDS range for that group. In addition, we compared leptin levels in children with adults with NW using selected data from a prepublished meta-analysis conducted by Chen et al (25). We extracted only those studies that reported leptin measurements for both men and women with normal weight. To visualize the sex differences in leptin levels across the pubertal stages in children and adults with NW and with children with OW/OB, we created a box plot using the ggplot2 package.

Considering the heterogeneity in the study populations in children with NW and with OW/OB, we used the random effects model to estimate the effect size of weighted mean difference. Based on the I2 index, the heterogeneity of the effect size was assessed. If the heterogeneity score was ≥ 75%, it was considered a high variation. For number of studies ≤5, Knapp-Hartung adjustments (26) were used to calculate the CIs around the effect size. Outliers’ analysis was performed to identify the extreme effect sizes in each of the subgroups. We conducted a sensitivity analysis to rule out the influence of any study on the pooled effect size after excluding the outliers. To assess the heterogeneity in leptin levels in children with NW, post hoc subgroup analysis was conducted to estimate the effect of sex differences in leptin according to the geographical region and study design. Funnels plots and Egger's regression were used to assess publication bias in both NW and OW/OB groups, when the number of studies was ≥ 6 (Figs. S1 and S2) (23). *P* ≤ .05 was considered statistically significant.

## Results

### Systematic Review

#### Study identification and selection

**Figure 1.**
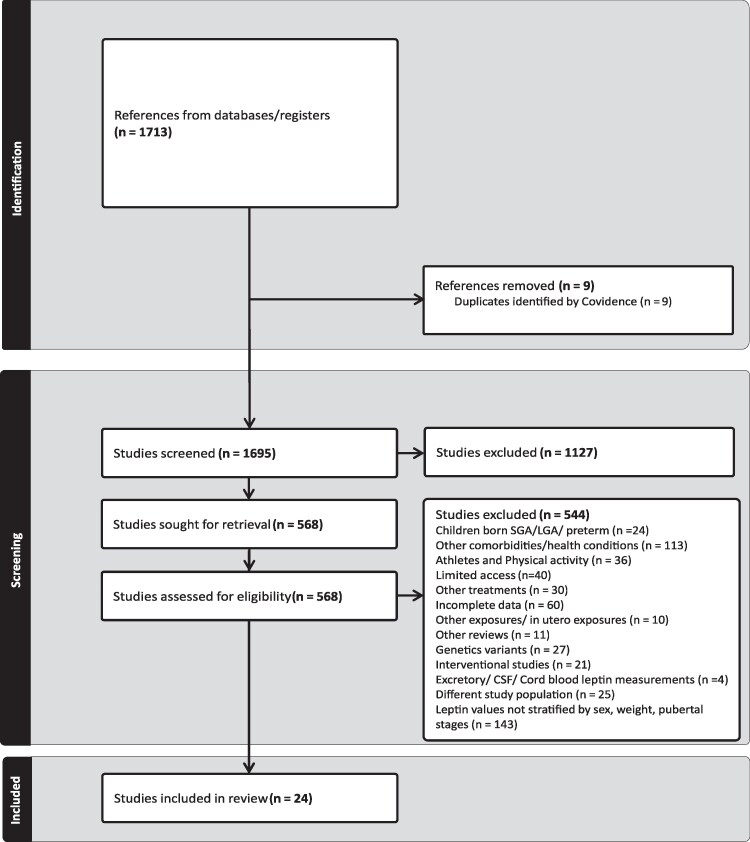
Systematic review flow chart.

The initial search strategy retrieved 1713 articles. After removal of duplicates, 1695 articles were included for title and abstract screening, resulting in 1127 articles being excluded and 568 articles included for full-text screening. Based on our inclusion and exclusion criteria, another 544 articles were excluded because the articles included children born small or large for gestational age or preterm (n = 24), children with comorbidities (n = 113), children performing physical activities (n = 36), children undergoing other treatments (n = 30), children subjected to intrauterine and other exposures (n = 10), or children with genetic variants (n = 27). Interventional studies (n = 21) and studies including a different study population (n = 25) were also excluded. In addition, studies evaluating leptin levels in urine and cerebrospinal fluid (n = 4) were excluded. We also excluded articles where leptin values were not differentiated for sex, weight, and pubertal stages (n = 143). Studies with incomplete data (n = 60), limited access (n = 40), and other reviews (n = 11) were also excluded. In total, 24 articles were included for the systematic review and meta-analysis (Fig. 1).

#### Characteristics of selected studies

The selected studies were divided into 2 categories: (1) children with NW (Table 1) and (2) children with OW/OB (Table 2). A total of 3588 girls and 3663 boys with NW and 476 girls and 435 boys with OW/OB were included in this systematic review to evaluate sex differences in leptin levels. The selected studies were performed in the United States (n = 8), Spain (n = 5), Germany (n = 1), Austria (n = 1), Sweden (n = 1), Denmark (n = 1), the United Kingdom (n = 1), Saudi Arabia (n = 1), Greece (n = 1), Italy (n = 1), Tunisia (n = 1), and Australia (n = 2) and were published in English. The studies comprised cross-sectional (n = 20), longitudinal cohort (n = 2) and case-control (n = 2) studies, with a sample size ranging from 11 to 839 children. For most of the studies, leptin levels were measured in blood samples drawn after fasting using ELISA (n = 7) or radioimmunoassay (RIA) (n = 15), electrochemiluminescence (n = 1) or multiplex immunoassay (n = 1), or antigenic immunoassay (n = 1) (Tables 1 and 2) (22, 27-49).

**Table 1.** Characteristics of selected studies including children with normal weight

Study	Country	Sample size		Age interval (years)		Puberty/Tanner stages	Girls		Boys		Assay
		Girls	Boys	Girls	Boys		Leptin (mean/median ± SD/SE/IQR) ng/mL	BMI/BMI-SDS (Mean ± SD)	Leptin (Mean ± SD/SE/IQR) ng/mL	BMI-SDS (Mean ± SD)	
Garcia-Mayor (27) 1997	Spain	188	327	8.37 ± 0.14	8.85 ± 0.12	Prepubertal	5.47 ± 0.28	0.131 ± 0.09	4.38 ± 0.22	0.439 ± 0.08	RIA
		49	34	11.22 ± 0.22	12.65 ± 0.19	Early pubertal	7.10 ± 0.71	0.575 ± 0.23	4.06 ± 0.46	0.355 ± 0.18	
		106	85	12.69 ± 0.13	13.38 ± 0.12	Pubertal	8.11 ± 0.55	0.631 ± 0.12	2.71 ± 0.19	0.602 ± 0.10	
Roemmich (22) 1998	USA	12	16	10.4 ± 0.3	10.4 ± 0.3	Prepubertal	11.3 ± 2.8	–—	7.1 ± 2.4	–—	RIA
		15	13	13.5 ± 0.3	13.4 ± 0.3	Pubertal	18.4 ± 2.5	–—	5.6 ± 2.6	–—	
Arslanian (28) 1998	USA	9	13	10.5 ± 0.2		Prepubertal	8.0 ± 1.5	–—	10.2 ± 3.2	–—	RIA
		14	13	13.5 ± 0.2		Pubertal	17.3 ± 3.5	–—	4.0 ± 0.5	–—	
Demerath (29) 1999	USA	27	29	9.5 ± 1.3	9.8 ± 1.5	Pre pubertal	6.7 ± 4.6	16.5 ± 2.0	4.7 ± 3.9	17.1 ± 2.1	RIA
		42	42	13.8 ± 1.9	13.6 ± 1.8	Pubertal	12.7 ± 8.1	19.6 ± 3.0	4.1 ± 4.1	19.1 ± 3.8	
		32	39	17.6 ± 0.9	17.7 ± 0.8	Post pubertal	19.6 ± 11.9	22.1 ± 2.8	4.2 ± 5.2	22.4 ± 5.1	
Horlick (30) 2000	USA	15	13	8.5 ± 0.3	8.7 ± 0.5	Tanner 1	3.9 ± 1.4	–—	9.1 ± 4.2	–—	ELISA
		8	9	10.6 ± 0.4	11.1 ± 0.4	Tanner 2	14.4 ± 4.4	–—	7.0 ± 2.6	–—	
		12	15	13.1 ± 0.5	13.6 ± 0.4	Tanner 3	11.6 ± 3.4	–—	14.6 ± 5.3	–—	
		8	6	16.8 ± 0.9	15.5 ± 0.7	Tanner 4	14.8 ± 5.3	–—	6.4 ± 3.3	–—	
		6	10	15.1 ± 0.9	16.7 ± 0.5	Tanner 5	30.3 ± 7.9	–—	3.5 ± 1.9	–—	
Danadian (31) 1999	USA	6	9	10.3 ± 0.2	9.7 ± 0.3	Prepubertal	9.5 ± 2.4	17.7 ± 1.2	4.4 ± 0.6	16.5 ± 0.3	RIA
		13	14	12.3 ± 0.4	13.04 ± 0.4	Pubertal	13.9 ± 3.0	21.2 ± 1.0	9.6 ± 3.6	23.0 ± 1.0	
Lloyd (32) 2010	USA	7	18	10.4 ± 0.7	11.4 ± 1.3	Prepubertal	8.2 ± 5.8	−0.13 ± 0.81	5.5 ± 4.3	0.21 ± 0.59	ELISA
		14	13	12.2 ± 1.2	13.8 ± 0.9	Pubertal	5.9 ± 3.4	−0.22 ± 0.61	2.2 ± 1.6	−0.30 ± 0.67	
		20	15	14.9 ± 1.4	16.0 ± 1.4	Post-pubertal	12.9 ± 7.3	0.28 ± 0.81	3.1 ± 2.2	0.21 ± 0.62	
Blum (33) 1997	Denmark	105	117	5.8 ± 19.7	6.0 ± 19.9	Tanner 1	2.51 (1.23–5.12)	16.30 ± 2.10	1.41 (0.65–3.04)	16.79 ± 2.03	RIA
		30	34			Tanner 2	2.86 (1.51–5.44)	16.94 ± 1.59	2.19 (0.91– 5.28)	18.91 ± 2.32	
		21	20			Tanner 3	3.81 (1.93–7.51)	18.11 ± 2.23	1.26 (0.48–3.29)	18.53 ± 2.90	
		21	29			Tanner 4	4.39 (2.67–7.23)	18.45 ± 1.63	0.79 (0.38– 1.64)	19.66 ± 1.73	
		156	111			Tanner 5	6.24 (3.57–10.89)	21.20 ± 2.39	0.71 (0.34–1.44)	21.25 ± 1.94	
Arrowsmith (34) 2002	Australia	12	14	7.9 ± 0.8	8.3 ± 0.8	Prepubertal	6.2 ± 3.7	0.0 ± 0.9	8.3 ± 5.6	0.5 ± 1.3	RIA*^a^*
		15	18	16.5 ± 0.5	17.0 ± 1.1	Pubertal	17.7 ± 12.0	0.0 ± 0.7	6.7 ± 9.1	0.4 ± 0.1	
Al-Daghri (35) 2011	Saudi Arabia	166	159	7.2 ± 11.3	11.9 ± 12.2	Prepubertal	11.9 ± 12.2	19.4 ± 4.7	7.2 ± 11.3	18.8 ± 5.2	ELISA
Byrnes (36) 1999	Australia	30	29	8.6 ± 0.2	8.5 ± 0.3	Prepubertal	11.5 ± 2.2	0.5 ± 0.3	6.5 ± 1.0	0.3 ± 0.1	RIA
Dencker (37) 2006	Sweden	79	91	9.89 ± 0.6	10.09 ± 0.6	Prepubertal	5.2 ± 4.8	17.4 ± 2.7	3.2 ± 4.2	17.5 ± 2.6	RIA*^a^*
Nagy (38) 1997	USA	23	20	7.3 ± 1.7	7.6 ± 1.6	Prepubertal	8.5 ± 9.9	18.6 ± 4.29	4.8 ± 5.6	19.0 ± 5.6	RIA
		8	23	7.6 ± 1.4	8.2 ± 1.5	Pre-pubertal	8.0 ± 6.1	18.8 ± 2.7	5.1 ± 5.6	17.9 ± 3.1	
Moore (39) 2004	UK	221	250	8.00 ± 0.68	8.01 ± 0.69	Tanner 1	2.36 ± 0.91	−1.42 ± 0.77	1.83 ± 0.47	−1.44 ± 0.92	RIA
Jmal (40) 2010	Tunisia	123	124	9.1 ± 1.39	9.51 ± 1.55	Prepubertal	2.44 ± 1.74	–—	1.91 ± 2.01	–—	RIA
		66	10	11.04 ± 0.83	10.94 ± 1.02	Early-pubertal	4.87 ± 3.55	–—	2.37 ± 2.85	–—	
Celi (41) 2005	Italy	770	980	8.5 (7.1–9.7)	9.3 (8.1–10.6)	Prepubertal	11.6 (5.3–19.5)	20.1 (16.1–23.9)	10.0 (4.1–18.4)	21.4 (16.2–24.8)	RIA
		976	727	11.1 (10.5–12.6)	11.9 (10.9–12.8)	Pubertal	15.6 (9.5–25.4)	22.9 (19.3–26.7)	9.0 (3.9–16.1)	23.3 (18.8–26.4)	

Abbreviations: BMI-SDS, body mass index SD score; IQR, interquartile range; RIA, radioimmunoassay.

^*a*^Double antibody radioimmunoassay.

**Table 2.** Characteristics of selected studies including children with overweight/obesity

Study	Country	Sample size		Age interval (years)		Puberty stages	Girls				Boys				Assay
		Girls	Boys	Girls	Boys		Leptin (mean/median ± SD/SE/IQR) ng/mL		BMI/BMI-SDS (mean ± SD)		Leptin (mean/median ± SD/SE/IQR) ng/mL		BMI/BMI-SDS (mean ± SD)		
							Obese	Control	Obese	Control	Obese	Control	Obese	Control	
Olza (42) 2014	Spain	104 NW	119 NW	8.78 ± 0.10	9.1 ± 0.10	Prepubertal	22.67 ± 1.34	3.87 ± 0.31	3.15 ± 0.11	0.22 ± 0.05	23.92 ± 1.38	4.21 ± 0.40	4.03 ± 0.12	−0.21 ± 0.05	Multiplex immunoassay
		104 OB	119 OB	8.57 ± 0.20	9.1 ± 0.10										
Sudi (43) 2000	Austria	13	32	9 ± 1.6	10.± 2.1	Prepubertal	15.5 ± 7.4	—	—	—	19.3 ± 19.7	—	—	—	RIA
		25	17	11.9 ± 1.2	13 ± 1.1	Pubertal	24.3 ± 10.9	—	—	—	20.1 ± 13.9	—	—	—	
		10	5	14.2 ± 1.1	14. ± 1.6	Postpubertal	28 ± 16.4	—	—	—	13.4 ± 10.4	—	—	—	
Jois (44) 2015	Spain	77 OW	53 OW			Prepubertal	27.2 (22.8–31.6)				16.7 (12.2–21.3)				ELISA
		33 OB	30 OB												
Maier (48) 2014	Germany	23 NW	25 NW	7.5 ± 1.2	7.5 ± 1.1	Prepubertal	13.8 ± 9.0	4.4 ± 3.2	1.85 ± 0.49	0.49 ± 0.66	10.2 ± 9.4	2.0 ± 1.6	2.03 ± 0.46	0.38 ± 0.48	ELISA
		57 OW	43 OW												
Stylianou (45) 2007	Greece	5 NW	10 NW	12.8 ± 1.11		Pubertal	33.52 ± 16.19	16.37 ± 5.86	—	—	35.97 ± 20.33	8.43 ± 5.06	—	—	RIA
		10 OB	10 OB	12.7 ± 1.87			47.26 ± 31.46				44.18 ± 20.06				
		14 OB- IR	6 OB- 1R	12.8 ± 1.82											
Rivera (49) 2019	Spain	30 OB	30 OB	6.03 (2.48)	7.89 (2.37)	Prepubertal	22.32 ± 8.92	—	4.93 ± 1.33	—	24.76 ± 13.13	—	5.60 ± 2.28	—	ELISA
		31 OB-IR	29 OB-IR	9.07 (1.93)	8.47 (1.67)		33.46 ± 11.00		4.78 ± 1.46		31.59 ± 13.19		5.46 ± 2.70		
Newbern (46) 2014	USA	41	41	14.0 ± 0.22	13.7 ± 0.26	Pubertal	63.10 ± 4.2	—	2.24 ± 0.05	—	42.34 ± 4.6		2.37 ± 0.06	—	ECL
Valle (47) 2005	Spain	31 NW	20 NW	7.74 ± 0.12		Pre-pubertal	16.21 ± 1.64	5.21 ± 0.89	—	—	14.59 ± 2.08	3.87 ± 0.79	—	—	ELISA
		31 OB	20 OB	7.63 ± 0.14											

Abbreviations: AIA, antigenic immunoassay; BMI-SDS, body mass index SD score; ECL, electrochemiluminescence assay; IQR, interquartile range; NW, normal weight; OB, obesity; OB-IR, obesity with insulin resistance; OW, overweight; PETIA, particle enhanced turbidimetric immunoassay; RIA, radioimmunoassay.

### Meta-analysis

#### Sex differences in leptin levels across pubertal stages in children with NW and children with OW/OB

**Figure 2.**
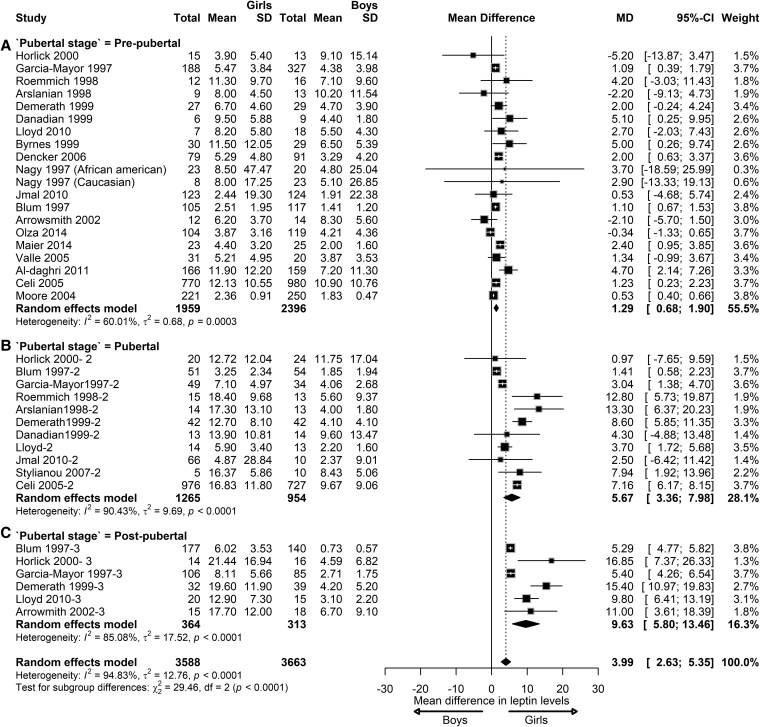
Sex differences in leptin levels in children with normal weight. Forest plot of the effect of sex in children with normal weight on leptin concentrations at prepubertal (A), pubertal (B), and postpubertal stages (C). Results are presented as mean difference (MD) and 95% CI, represented by the black square and black horizontal line, respectively. The black diamond in all 3 groups along with the vertical dashed line represents the estimated overall effect size of all studies. The width of the diamond represents the overall pooled 95% CI.

In children with NW, the meta-analysis indicated a significant sex difference in leptin levels. Girls had higher leptin levels compared to boys, with a pooled effect of 3.99 ng/mL (95% CI, 2.63-5.35; *I*^2^ = 94.83%; *P* < .01) (Fig. 2). Significant differences were observed between pubertal stages (*P* < .01). At the prepubertal stage, girls had 1.29 ng/mL higher leptin levels than boys (95% CI, 0.68-1.90; *I*^2^ = 60.01%; *P* < .01). During the pubertal stage, leptin levels in girls were 5.67 ng/mL higher compared to boys (95% CI, 3.36-7.98; *I*^2^ = 90.43%; *P* < .01). At the postpubertal stage, this difference further increased with girls having 9.63 ng/mL higher leptin levels than boys (95% CI, 3.38-14.00; *I*^2^ = 99.33%; *P* < .01).

**Figure 3.**
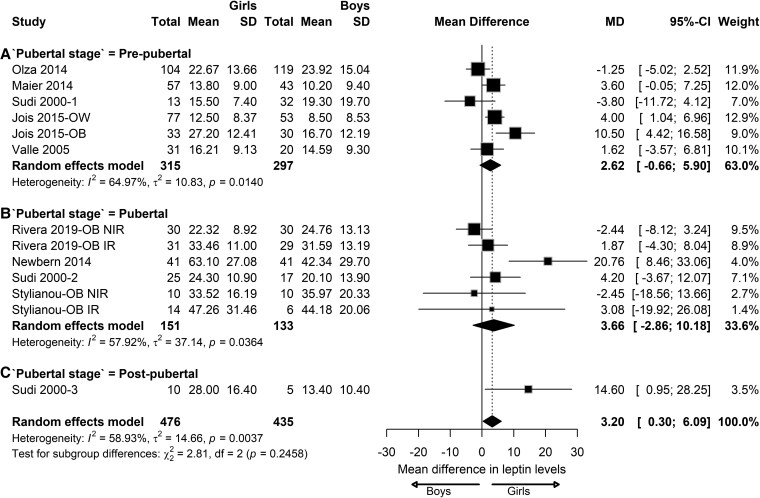
Sex differences in leptin levels in children with overweight/obesity. Forest plot of the effect of sex in children with overweight/obesity on BMI-SDS at prepubertal (A), pubertal (B), and postpubertal (C) stages. Results are presented as mean difference (MD) represented by the black square and 95% CI represented by the black horizontal line. The black diamond in all 3 groups along with the vertical dashed line represent the estimated overall effect size of all studies. The width of the diamond represents the overall pooled 95% CI.

In children with OW/OB, leptin levels increase significantly in both boys and girls and the pooled effect showed significant sex differences in leptin levels (mean difference [MD]: 3.16 ng/mL; 95% CI, 0.29-6.03) (Fig. 3). No significant differences were observed in leptin levels between girls and boys at the prepubertal and pubertal stages. Insignificant differences were observed between the pubertal stages (*P* = .24). At the postpubertal stage, only 1 study could be analyzed, reporting 14.60 ng/mL higher leptin levels in girls compared to boys (95% CI, 0.95-28.25).

**Figure 4.**
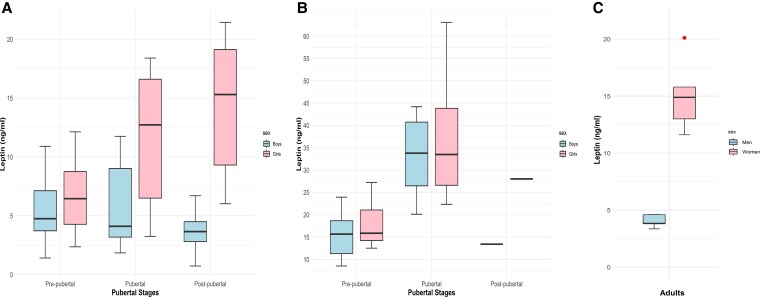
Box plot illustrating leptin levels in girls and boys with (A) normal weight and (B) with overweight/obesity during prepubertal, pubertal, and postpubertal stages. Box plot illustrating leptin levels in men and women with normal weight (C). Data are presented as mean and interquartile range, with the horizontal line in each bar representing the median leptin level.

In children with NW and OW/OB, the box plots illustrate the mean differences in leptin levels across pubertal stages in boys and girls (Fig. 4A and 4B). Analysis of leptin levels in NW adults, extracted from the study of Chen et al (25), shows that the sex difference in leptin levels persists in adulthood (Fig. 4C). These data also show that postpubertal leptin levels in boys and girls with NW are comparable to those in adult men and women, whereas those in children with OW/OB exceed adult levels (Fig. 4C).

#### Sex differences in BMI-SDS in children with NW and children with OW/OB

**Figure 5.**
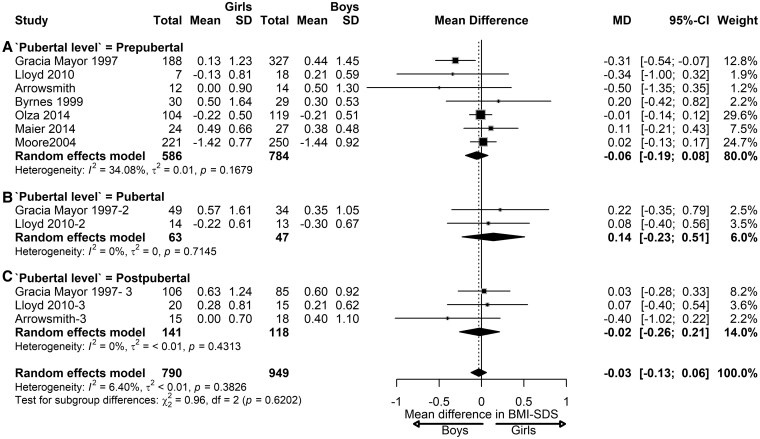
Sex differences in BMI-SDS in children with normal weight. Forest plot of the effect of sex in children with normal weight on BMI-SDS at prepubertal (A), pubertal (B), and postpubertal stages (C). Results are presented as mean difference (MD) represented by the black square and 95% CI represented by the black horizontal line. The black diamond in all the 3 groups along with the vertical dashed line represent the estimated overall effect size of all studies. The width of the diamond represents the overall pooled 95% CI.

In children with NW, the pooled effect showed no significant sex differences in BMI-SDS (MD = −0.03; 95% CI, −0.13 to 0.06; *I*^2^ = 6.40%%; *P* = .38) (Fig. 5). No significant differences were observed between pubertal stages. Girls tended to have slightly lower BMI-SDS than boys in prepubertal (MD = −0.06; 95% CI, −0.19 to 0.08) and similar BMI-SDS at the pubertal (MD = 0.14; 95% CI, −0.23 to 0.51; *I*^2^ = 6%; *P* = 0.71) and postpubertal stages (MD = −0.02; 95% CI, −0.26 to 0.21).

**Figure 6.**
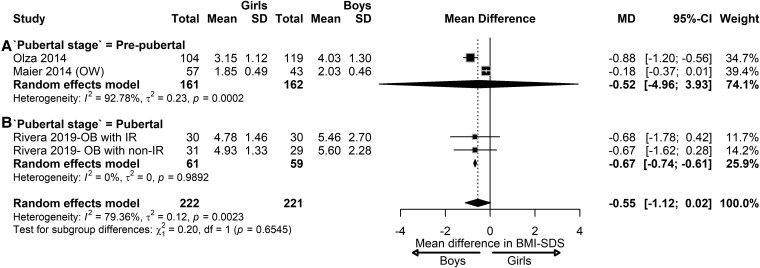
Sex differences in BMI-SDS in children with overweight/obesity. Forest plot of the effect of sex in children with overweight or obesity on BMI-SDS at prepubertal (A) and pubertal stage (B). Results are presented as mean difference (MD) represented by the black square and 95% CI represented by the black horizontal line. The black diamond in all 3 groups along with the vertical dashed line represent the estimated overall effect size of all studies. The width of the diamond represents the overall pooled 95% CI.

In children with OW/OB, the pooled effect showed no significant differences in BMI-SDS (MD = −0.55; 95% CI, −1.12 to 0.02 (Fig. 6). However, based on this limited number of studies, at the prepubertal stage, girls tended to have a 0.52 lower BMI-SDS score than boys, although this difference was insignificant (95% CI, −4.96 to 3.93; *I^2^* = 92.78%; *P* < .01), and at the pubertal stage, girls had a significantly lower BMI-SDS score compared to boys (MD = −0.67; 95% CI, −0.74; −0.61). No studies were identified to analyze sex differences in BMI-SDS at the postpubertal stage.

### Post hoc Analysis

#### Sex differences in leptin levels in children with NW according to geographical region

**Figure 7.**
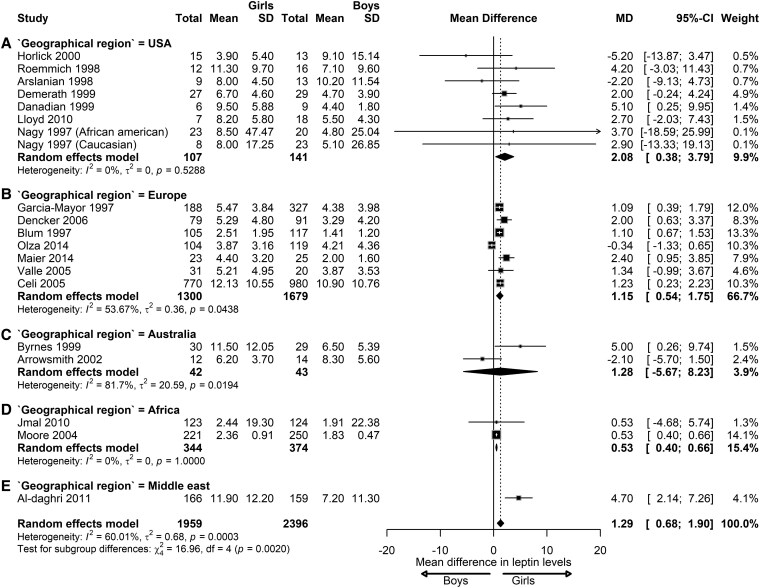
Sex difference in leptin levels in prepubertal children with normal weight according to geographical region. Forest plot of sex differences in leptin levels in children with normal weight at the prepubertal stage from the geographical regions: (A) United States (B), Europe (C), Australia (D), Africa (E), and the Middle East. Results are presented as mean difference (MD) represented by the black square and 95% CI represented by the black horizontal line. The black diamond in all 3 groups along with the vertical dashed line represent the estimated overall effect size of all studies. The width of the diamond represents the overall pooled 95% CI.

To analyze whether the geographical region of the selected studies influenced leptin levels, we performed subgroup analysis. The majority of the studies included were performed in the United States or Europe. In the US analyzed cohorts of NW children, leptin levels were 2.08 ng/mL higher in girls than boys at the prepubertal stage (MD = 2.08; 95% CI, 0.38-3.79; *I*^2^= 0%; *P* = .5) (Fig. 7). In studies performed in NW children across Europe, leptin levels were 1.15 ng/mL higher in girls than boys (MD = 1.15; 95% CI, 0.54-1.75; *I*^2^= 53.67%; *P* = .04) (Fig. 7). For the other geographical regions, for which only 1 (Middle East) or 2 (Australia and Africa) studies were available, leptin levels were reported to be higher in girls than in boys: Middle East (MD = 4.70; 95% CI, 2.14-7.26), Australia (MD = 1.28; 95% CI, −5.67 to 8.23), and Africa (MD = 0.53; 95% CI, 0.44-0.66; *I*^2^= 0%; *P* = 1). Significant subgroup differences were observed between geographical regions (Fig. 7).

**Figure 8.**
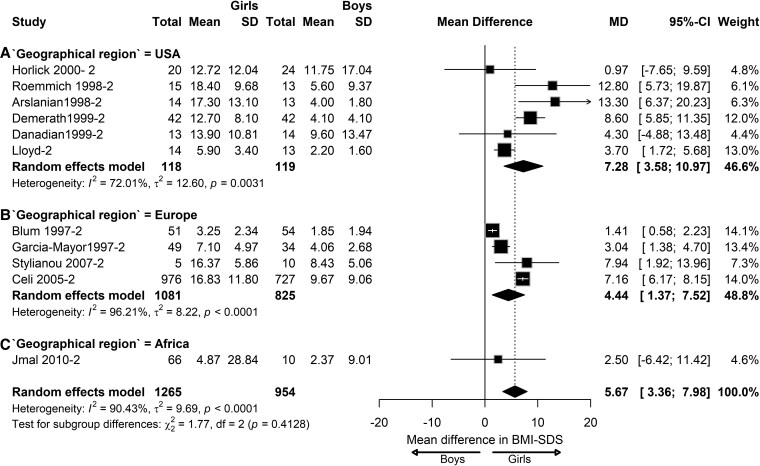
Sex difference in leptin levels in pubertal children with normal weight according to geographical region. Forest plot of sex differences in leptin levels in children with normal weight at the postpubertal stage from the geographical regions: (A) United States, (B) Europe, and (C) Africa. Results are presented as mean difference (MD) represented by the black square and 95% CI represented by the black horizontal line. The black diamond in all 3 groups along with the vertical dashed line represent the estimated overall effect size of all studies. The width of the diamond represents the overall pooled 95% CI.

At the pubertal stage, leptin levels were 7.28 ng/mL higher in girls than boys with NW of US cohorts (95% CI, 3.58-10.97; *I*^2^= 72.01%; *P* = .0031) (Fig. 8). In European studies, leptin levels were 4.44 ng/mL higher in girls compared to boys (95% CI, 1.37-7.52; *I*^2^= 96.21%; *P* < .0001). Only 1 study was available for an African cohort, showing no significant sex differences in leptin levels (MD = 2.50; 95% CI, −6.42 to 11.42). No significant subgroup differences were observed between geographical regions (Fig. 8).

**Figure 9.**
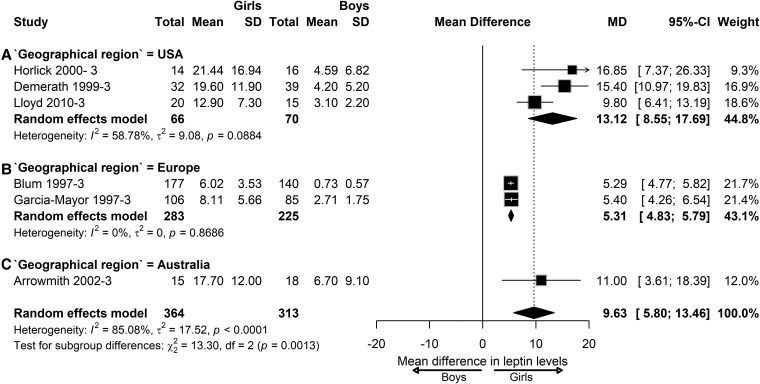
Sex difference in leptin levels in postpubertal children with normal weight according to geographical region. Forest plot of sex differences in leptin levels in children with normal weight at the postpubertal stage from the geographical regions: (A) United States, (B) Europe, and (C) Australia. Results are presented as mean difference (MD) represented by the black square and 95% CI represented by the black horizontal line. The black diamond in all 3 groups along with the vertical dashed line represent the estimated overall effect size of all studies. The width of the diamond represents the overall pooled 95% CI.

For the postpubertal stage, only studies from the United States (n = 3), Europe (n = 2), and Australia (n = 1) were available. Leptin levels were significantly higher in girls than boys of US cohorts (MD = 13.12; 95% CI, 8.55-17.69; *I*^2^= 58.78%; *P* = .08) (Fig. 9). In NW European children, leptin levels were found to be 5.31 ng/mL higher in girls than in boys (95% CI, 4.83-5.79; *I*^2^= 0%; *P* = .86). For Australia, only 1 study was available that showed significantly higher leptin levels than boys (MD = 11.00; 95% CI, 3.61-18.39). Significant subgroup differences were observed between geographical regions (Fig. 9).

In summary, a larger sex difference in leptin levels was observed in the US cohorts with NW, with girls having higher leptin levels than boys, suggesting a potential geographical influence on leptin. There were no eligible studies from Asia included in our analysis.

#### Sex differences in leptin levels in children with NW according to study design

**Figure 10.**
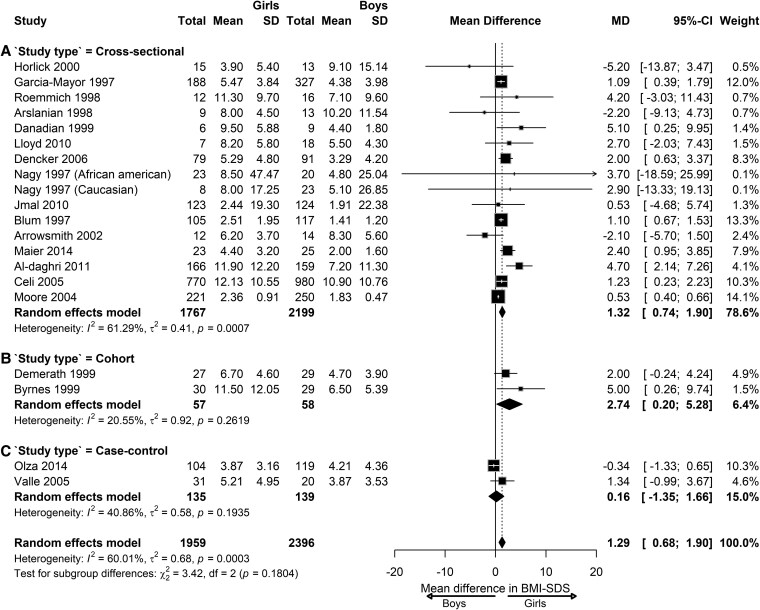
Sex difference in leptin levels in prepubertal children with normal weight according to the study design. Forest plot of sex differences in leptin levels in children with normal weight at the prepubertal stage in the following studies: (A) cross-sectional, (B) cohort, and (C) case control. Results are presented as mean difference (MD) represented by the black square and 95% CI represented by the black horizontal line. The black diamond in all 3 groups along with the vertical dashed line represent the estimated overall effect size of all studies. The width of the diamond represents the overall pooled 95% CI.

**Figure 11.**
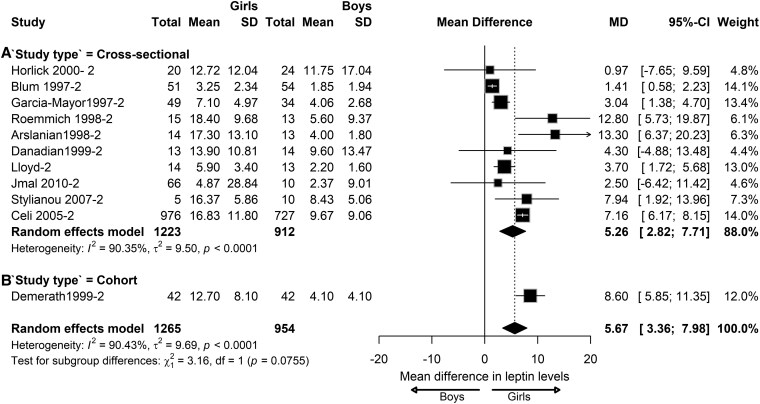
Sex difference in leptin levels in pubertal children with normal weight according to the study design. Forest plot of sex differences in leptin levels in children with normal weight at the pubertal stage in the following studies: (A) cross-sectional and (B) cohort. Results are presented as mean difference (MD) represented by the black square and 95% CI represented by the black horizontal line. The black diamond in all 3 groups along with the vertical dashed line represent the estimated overall effect size of all studies. The width of the diamond represents the overall pooled 95% CI.

**Figure 12.**
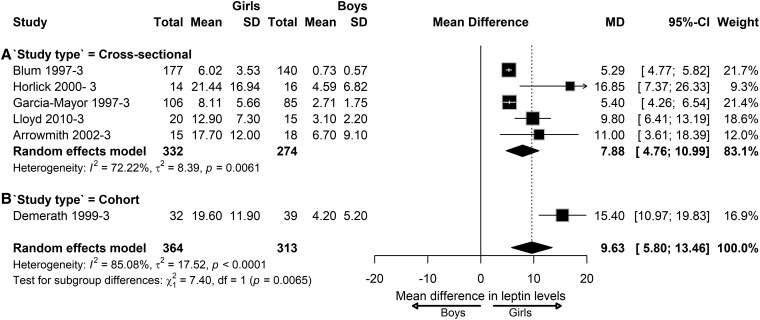
Sex difference in leptin levels in post-pubertal children with normal weight according to the study design. Forest plot of sex differences in leptin levels in children with normal weight at the postpubertal stage in the following studies: (A) cross-sectional or (B) cohort. Results are presented as mean difference (MD) represented by the black square and 95% CI represented by the black horizontal line. The black diamond in all 3 groups along with the vertical dashed line represent the estimated overall effect size of all studies. The width of the diamond represents the overall pooled 95% CI.

To analyze whether the study design influenced the sex differences in leptin levels, we also performed subgroup analysis according to the study design in children with NW. At the prepubertal stage, we found that the sex differences in leptin levels varied across cross-sectional studies (MD = 1.32; 95% CI, 0.74-1.90; *I^2^ =* 61.29%; *P* = .0007), cohort studies (MD = 2.74; 95% CI, 0.20-5.28; *I^2^ =* 20.55%; *P* = .26), and case-control studies (MD = 0.16; 95% CI, −1.35 to 1.66; *I^2^ =* 40.86%; *P* = .19), although insignificantly (Fig. 10). At the pubertal stage, the sex differences in leptin levels varied across cross-sectional studies (MD = 5.26; 95% CI, 2.82-7.71; *I^2^ =* 90.35%; *P* < .0001) and cohort studies (MD = 8.60; 95% CI, 5.85-11.35), although only 1 cohort study was available for analysis. No significant subgroup differences were observed between the 2 study designs (Fig. 11). At the postpubertal stage, the sex differences in leptin levels varied significantly across cross-sectional (MD = 7.88; 95% CI, 4.76-10.99; *I^2^ =* 72.22%; *P* = .0061) and cohort study designs (n = 1) (MD = 15.40; 95% CI, 10.97-19.83; *I^2^ =* 85.08%; *P* < .0001) (Fig. 12).

Overall, despite slight variations in sex differences in leptin levels across different study designs, no significant influence of study design on sex differences in leptin levels was observed.

### Sensitivity Analysis

#### Children with NW

We found high heterogeneity in all studies addressing leptin levels at the pubertal and postpubertal stages in children with NW (*I*^2^ > 75%). However, most of the individual studies demonstrated higher leptin levels in girls compared to boys. The studies of Olza et al, Al-daghri et al, and Moore et al were identified as outliers for the prepubertal stage (35, 39, 42). Upon removal of these studies, the effect size changed slightly from 1.29 to 1.27, but also reduced the heterogeneity from 60.01% to 6.2% (95% CI, 0.93-1.56; *P* < .0001). Similarly, at the pubertal stage, the study of Blum et al was identified as an outlier (33). Excluding this study resulted in an increased effect size (5.67 to 6.30) and decreased heterogeneity (90.43% to 75.3%). No outliers were found in the postpubertal subgroup. Influential analysis showed that omitting the study of Moore et al significantly reduced the heterogeneity from 60.01% to 46% (39). At the pubertal stage, omission of the study of Blum et al resulted in a reduction of heterogeneity from 90.4% to 75.3%, whereas at the postpubertal stage, the heterogeneity was reduced from 85.08% to 72% after omitting the study of Demerath et al from the analysis (29, 33) (Table S3A, B, and C, respectively) (23).

#### Children with OW/OB

We found moderate heterogeneity in leptin levels in children with OW/OB (*I*^2^ < 20%-70%). No outliers were found in the prepubertal and postpubertal subgroups. However, the influential analysis showed a significant reduction of heterogeneity from 64.97% to 55.1% upon omitting the study of Olza et al from the prepubertal subgroup (42). Omitting the study of Newbern et al from the pubertal subgroup showed a reduction of heterogeneity from 57.2% to 0%, although this reduction was statistically insignificant (46) (Table S4A and S4B, respectively) (23).

## Discussion

This systematic review and meta-analysis describes sex differences in leptin levels across pubertal status in children and adolescents with NW and with OW/OB. We found that sex differences in leptin levels are present at all pubertal stages in children with NW. However, in children with OW/OB, this sex difference in prepubertal children disappeared completely at the pubertal stage, whereas postpuberty, leptin levels differed again between boys and girls, although the latter is based on 1 study with a small sample size. Therefore, the findings in postpubertal children with OW/OB should be interpreted with caution before generalizing them across geographical regions and ethnic groups.

In children with NW, girls had higher leptin levels than boys. This sex difference in leptin levels tended to augment during pubertal development. A specific leptin threshold is required for the pubertal (re)activation of the HPG axis via kisspeptin neurons, which causes the release of sex steroids (50). Although leptin does not seem to play a role in the initial neonatal activation of the HPG axis referred to as mini-puberty, it is considered a metabolic trigger for the reactivation of the HPG axis during adolescence (51). However, sex steroids in turn regulate leptin levels creating a negative feedback loop between metabolic and reproductive function (8, 9). Estradiol stimulates leptin secretion, contributing to higher leptin levels in girls, while testosterone inhibits its secretion, leading to lower levels in the boys (21). Hence it is plausible that these sex differences in leptin levels, particularly postpuberty, are driven by the secondary effects of the sex steroids, and the sex differences in leptin observed in children with NW may not be the cause, but rather a consequence of sex-specific pubertal mechanisms.

In comparison to our current meta-analysis findings in children with NW, we observed that leptin levels were generally higher in both girls and boys with OW/OB, a result that also corresponds with findings from other studies (42, 48, 52, 53). This suggests that in children with OB, leptin levels reach a threshold much earlier in the prepubertal stage, potentially altering pubertal mechanisms. Previous research indicates that elevated leptin levels are linked with early puberty in girls with obesity, but this is less pronounced for boys (54, 55). Leptin acts through the kisspeptin system in the hypothalamus to regulate puberty onset. Interestingly, in addition to leptin, kisspeptin levels show a sex dimorphic pattern. Not only do levels increase earlier in girls compared to boys during pubertal development, peripheral kisspeptin levels are also elevated in prepubertal girls with OW or OB (56-58). Furthermore, also in the hypothalamus, girls exhibited a higher number of kisspeptin neurons and higher kisspeptin levels than boys (56, 59, 60). While leptin directly stimulates kisspeptin expression, the majority of kisspeptin neurons do not express leptin receptors, which suggest that there are also leptin-independent mechanisms regulating kisspeptin (56, 59, 61). The regulation of GnRH pulsatility in the onset of puberty is a complex mechanism, in which kisspeptin neurons in the arcuate nucleus (ARC) are considered to play a crucial role. Within the ARC, kisspeptin release is coordinated by neurokinin B as a positive regulator and dynorphin A as a negative regulator, both coexpressed with kisspeptin in the ARC. Furthermore, POMC neurons, expression α-melanocortin-stimulating hormone, and Agouti-related peptide (AgRP) neurons, expressing AgRP and neuropeptide Y, also provide metabolic control of kisspeptin neurons in the ARC of the hypothalamus. Because POMC and AgRP neurons are regulated by leptin, this provides an additional indirect manner of leptin to regulate kisspeptin release. The hypothalamic control of puberty and the role of kisspeptin are extensively reviewed by Jimenez-Puyer et al (62).

These studies suggest that kisspeptin neurons may provide an additional mechanism integrating metabolic status and puberty timing (63). The high leptin levels, combined with sex dimorphism in kisspeptin levels, potentially explain why elevated leptin levels lead to early puberty in girls with OB but less so in boys with OB.

This supports the hypothesis that while leptin plays a permissive role in pubertal initiation, kisspeptin acts as a main trigger for the pubertal timing. Interestingly, in boys with OB, the elevated leptin levels follow a female-like pattern with an increase, instead of decline, which is seen in boys of NW, during puberty. Leptin receptors are expressed at all levels of the HPG axis, including ovarian granulosa, theca cells, and testicular Leydig cells (64-66). Indeed, research suggests that elevated leptin levels inhibit testosterone secretion by the testicular Leydig cells (67). Similarly, leptin can also inhibit estrogen production, suggesting that leptin can also directly regulate gonadal steroidogenesis independent of the HPG axis (68). Indeed, animal studies suggest that leptin has a direct inhibitory role on testosterone secretion (69). However, it should be noted that this inhibitory effect was observed only in adult rats and not in prepubertal rats, independent of their nutritional status (69). Additionally, increased adiposity facilitates the conversion of androgens to estrogen via aromatase activity in adipocytes, which is stimulated by leptin, thereby leading to lower circulating testosterone levels and potentially delaying pubertal initiation in boys, as has been suggested for boys with severe obesity (60, 70-72).

Our results in children with NW also align with studies not included in our analysis, assessing leptin levels across all stages of puberty, which demonstrated that leptin levels continue to increase in girls, whereas in boys, leptin levels either decrease after Tanner stage 2 or remain relatively stable (73, 74). Additionally, a significant sex difference was observed at the postpubertal stage after adjusting for BMI and percentage body fat (27, 29, 30, 33). Indeed, we observed that the mean difference in leptin levels was highest at the postpubertal stage. During puberty, increased levels of testosterone cause changes in body composition in boys, including decreased fat mass and increased muscle mass. This may subsequently result in lower leptin levels in boys compared to girls, as also suggested by studies conducted by Blum et al and Clayton et al (33, 74). Also, in adults, leptin levels remain significantly higher in pre- and postmenopausal women compared to men, regardless of correcting for fat mass (75). Indeed, our analysis of data extracted from a meta-analysis by Chen et al confirmed that these sex differences observed postpuberty persist in adulthood (25). Findings from Xu et al suggest that both leptin and BMI increase gradually during pubertal development, in both boys and girls, with a steady increase in fat mass observed only in girls (76). However, no such trend was observed for BMI-SDS in our analysis performed in the group with NW. Only at the pubertal stage did girls have a slightly higher BMI-SDS than boys. However, it should be noted that only a few studies could be included in our meta-analysis of BMI-SDS.

In children with OW/OB, we found that the BMI-SDS was significantly higher in boys, compared to girls at prepubertal and pubertal stages (14). A critical BMI threshold is required for the onset of puberty in both girls and boys (14). However, this threshold is significantly higher for boys compared to the girls. This could likely be attributed to sex-specific actions of leptin on pubertal development. Although girls with OW/OB are more prone to early puberty because of the direct action of leptin on HPG axis via kisspeptin, the pubertal timing in boys with OW/OB seems to be either less affected or delayed by the elevated leptin levels because of a potentially different mechanism mediated via kisspeptin or at the level of gonadal sex steroid production, as discussed previously. On the other hand, it is known that obesity is associated to central resistance to leptin (77). Interestingly, a study in rats suggests that females displayed greater and sustained responses to centrally leptin overexpression, whereas males rapidly developed leptin resistance (78). Whether this sex difference in leptin sensitivity can easily be translated to human remains to be determined. To our knowledge, there are currently no methods to determine leptin sensitivity in a clinical setting. Furthermore, in addition to cellular resistance, leptin resistance can also develop due to impaired transport across the blood-brain barrier (77). These mechanisms complicate interpretation of leptin levels in obesity in relation to puberty onset.

We conducted a post hoc subgroup analysis of all the studies according to geographical region and study design to evaluate the cause of heterogeneity in sexual dimorphism in leptin levels in children with NW. We observed that the sex difference in leptin levels was higher in children from the United States compared to Europe. However, these differences were only statistically significant at the pubertal and postpubertal stages. The higher BMI-SDS values in children of the US cohorts likely explain this larger difference. However, firm conclusions cannot be drawn because of the low number of studies included and low number of participants in those US cohorts. In subgroup analysis according to study design, the influence of study design was not profound. However, these results should be interpreted with caution due to high variance in the leptin levels found within these subgroups.

This study has several limitations. There was a moderate to high heterogeneity in leptin levels at each pubertal stage. This heterogeneity could be due to the use of different assays, such as ELISA and RIA, contributing to variations in leptin levels. However, while differences in the range of leptin levels between assay types cannot be excluded, previous studies comparing leptin RIA and ELISA showed a high correlation (79, 80). Of the included studies in this meta-analysis, RIA was more frequently used compared to ELISA (n = 13 and n = 3, respectively) to assess leptin levels in children of NW, whereas for children with OW/OB, this was reversed (n = 2 and n = 4, respectively). However, analyzing only those studies using RIA or ELISA did not impact the findings (results not shown). We combined OW and OB to assess leptin levels that contributed to variations in leptin levels in the group with OB. Additionally, we only included children undergoing normal pubertal development and cannot draw any associations between leptin levels and pubertal onset. Also, smaller number of studies were available for the prepubertal and pubertal groups for the analysis of sex differences in BMI-SDS in children with OW or OB, making it difficult to draw firm conclusions. Although pubertal development was assessed using Tanner staging in most of the included studies, some of the studies used self-validated questionnaires. This may raise concerns about accuracy and potential biases, as children might tend to overestimate and underestimate their pubertal staging depending on age (81). These limitations highlight the need for more longitudinal studies assessing leptin and other (steroidal) hormones involved in the pubertal process, particularly in children with OB.

In conclusion, this systematic review and meta-analysis sheds light on the relationship between leptin levels and puberty in children with NW and those with OW/OB. It mainly emphasizes on the impact of obesity and leptin levels on sex-specific pubertal mechanisms. It also highlights sex differences in the pubertal timing and metabolic changes that occur during puberty. Our study suggests that the earlier puberty found in girls with obesity but not in boys cannot be explained by higher leptin levels. This study therefore can contribute to understanding the importance of leptin as a pubertal and obesity marker in the pubertal timing while highlighting the need to understand the sex-specific mechanisms involved in the link between obesity and puberty.

## Data Availability

Some or all datasets generated during and/or analyzed during the current study are not publicly available but are available from the corresponding author on reasonable request.

## Abbreviations

AgRP: Agouti-related peptide
ARC: arcuate nucleus
BMI-SDS: body mass index SD score
HPG: hypothalamus-pituitary-gonadal
MD: mean difference
NW: normal weight
OB: obesity
OW: overweight
POMC: pro-opiomelanocortin
RIA: radioimmunoassay
